# MicroRNA-26b attenuates monocrotaline-induced pulmonary vascular remodeling via targeting connective tissue growth factor (CTGF) and cyclin D1 (CCND1)

**DOI:** 10.18632/oncotarget.10125

**Published:** 2016-06-17

**Authors:** Ran Wang, Xing Ding, Sijing Zhou, Min Li, Li Sun, Xuan Xu, Guanghe Fei

**Affiliations:** ^1^ Department of Respiratory Medicine, The First Affiliated Hospital of Anhui Medical University, Hefei 230022, China; ^2^ Department of Occupational Diseases, Hefei Third Clinical College, Anhui Medical University, Hefei 230001, China; ^3^ Department of Oncology, The First Affiliated Hospital of Anhui Medical University, Hefei 230022, China; ^4^ Division of Pulmonary/Critical Care Medicine, Cedars Sinai Medical Center, Los Angeles, CA 90048, USA

**Keywords:** miR-26b, CTGF, cyclin D1, monocrotaline, pulmonary hypertension

## Abstract

MicroRNAs are involved in the control of cell growth, and deregulated pulmonary artery smooth muscle cell proliferation plays an essential role in the development of pulmonary hypertension. The objective of this study was to identify differentially expressed microRNA(s) and explore its therapeutic role in treatment of the disease. MicroRNA expression profile analysis showed microRNA-26b was differentially expressed in pulmonary artery smooth muscle cells harvested from monocrotaline-treated rats, and we validated microRNA-26b targets, in vitro and in vivo, CTGF and CCND1, both of which have been shown, in our previous work, to be involved in the pathogenesis of pulmonary hypertension. In vivo experiments demonstrated monocrotaline-induced pulmonary artery remodeling could be almost completely abolished by administration of microRNA-26b, while CTGF or CCND1 shRNA significantly, but only partially, attenuated the remodeling by silencing the designed target. Additionally, exogenous expression of the microRNA-26b substantially downregulated CTGF and CCND1 in human pulmonary artery smooth muscle cells. MicroRNA-26b might be a potent therapeutic tool to treat pulmonary hypertension.

## INTRODUCTION

Pulmonary artery hypertension (PAH) is a life-threatening medical condition of lungs characterized by continuous vasoconstriction, refractory elevation of pulmonary arterial pressure, as well as the pathogenic hallmark of vascular remodeling that primarily involves smooth muscle layer of the vessel wall [1, 2]. Abnormal proliferation of pulmonary artery smooth muscle cells (PASMCs) with the extension of smooth muscle into small, normally nonmuscular pulmonary arteries leads to medial muscularization and hypertrophy, resulting in obliteration and ultimately the obstruction of precapillary pulmonary arteries, and sustained elevation of pulmonary arterial pressure [3].

Our previous studies have demonstrated that connective tissue growth factor (CTGF) and cyclin D1 (CCND1) were evidently involved in the molecular mechanism underlying cigarette smoke-induced pulmonary vascular remodeling by promoting rat PASMCs (rPASMCs) proliferation [4, 5]. CTGF is a 38 kDa, cysteine-rich protein that was believed to be involved in the regulation of various cellular activities such as adhesion, migration, proliferation, extracellular matrix (ECM) synthesis in a wide spectrum of cell types, including vascular endothelial cells, fibroblasts, epithelial cells and smooth muscle cells [6–10]. CCND1 is a critical member of cyclin family, regulating the progression of the cell cycle, and plays a key role in controlling G1/S transition [11, 12]. Recently, a growing body of evidence indicated that CTGF plays a critical role in the regulation of cell cycle progression [13, 14], and it has been reported that CCND1, as a ‘mitogenic sensor’, mediated CTGF-induced proliferation of PASMCs as well as other types of cells [15, 16] by driving target cells through the restriction point in the G1 phase of their cycle [17]. It has been well documented that both CTGF and CCND1 were subject to the regulation of microRNA in lungs as well as vascular tissues [18-21].

MicroRNA (miRNA) is a class of ~22-nucleotide-long non-protein-coding RNAs, which can regulate gene expression by binding 3′ untranslated region (UTR) of target gene messenger RNA (mRNA), resulting in translational repression and/or mRNA degradation [22]. It is generally believed that approximately 1/3 human genes can be regulated by miRNA [23], and miRNA plays a key role in cellular activities such as cell proliferation, migration, and apoptosis [24-26]. To elucidate how miRNAs are involved in PAH pathogenesis, we investigated and compared global miRNA expression profiles in PASMCs isolated from monocrotaline-treated rats and normal control using microarray and selected the most significantly down-regulated miRNAs, miR-26b, for further functional analysis due to its documented role in regulating human cell proliferation [27], as well as the virtual function to suppress the expression of CTGF and CCND1 predicted by online microRNA databases such as www.targetscan.org.

In our previous studies, we showed that intratracheal administration of CTGF or CCND1 shRNA could significantly, but only partially, restore monocrotaline-induced pulmonary artery remodeling [28, 29]. In the present study, we found that downregulation of miR-26b was responsible for the upregulation of CTGF and CCND1 in monocrotaline-induced pulmonary artery remodeling, and intratracheal administration of miR-26b could almost completely restore the pulmonary artery remodeling in monocrotaline-treated rats.

## RESULTS

### Screening of differentially expressed miRNA between monocrotaline-treated rats and controls

**Figure 1:**
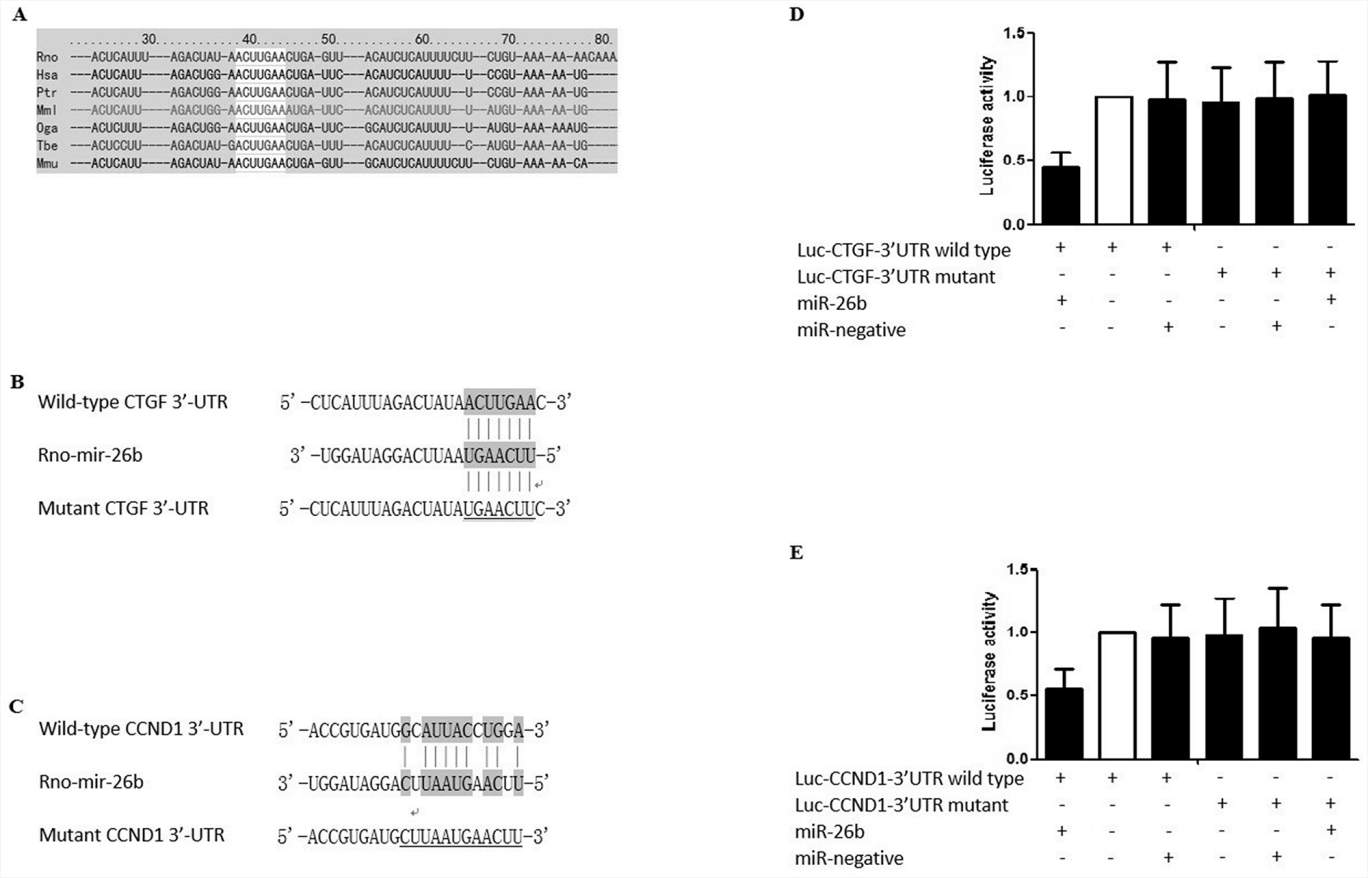
Sequence comparison between miR-26b and 3′UTR of CTGF and CCND1 **A.** The “seed sequence” in the 3′ UTR of the target gene of rno-miR-26b is highly conserved among the species including, but not limited to, Rno, Has, Ptr, Mml, Oga, Tbe, and Mmu; **B.** Schematic comparison between rno-miR-26b and the wild-type (upper sequence)/mutated (lower sequence) 3′UTR of CTGF with “seed sequence” highlighted; **C.** Schematic comparison between rno-miR-26b and the wild-type (upper sequence)/mutated (lower sequence) 3′UTR of CCND1 with “seed sequence” highlighted; **D.** The relative luciferase activity in the rPASMCs transfected with both wild-type 3′UTR of CTGF and rno-miR-26b is significantly lower than the controls (p<0.01); **E.** The relative luciferase activity in the rPASMCs transfected with both wild-type 3′UTR of CCND1 and rno-miR-26b is significantly lower than the controls (p<0.01). All experiments were repeated three times (N value=3).

**Figure 2:**
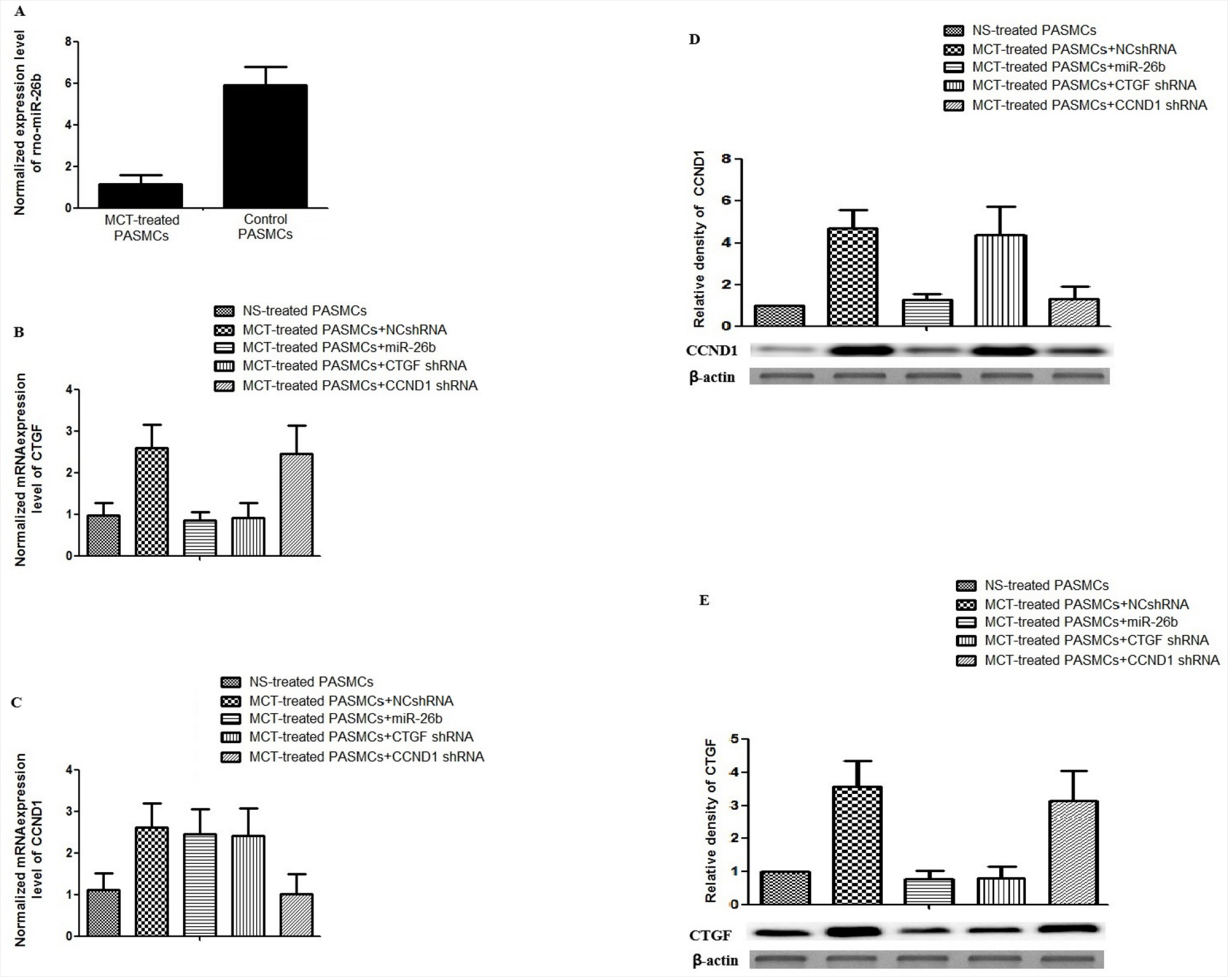
Effect of MCT, CTGF shRNA, CCND1 shRNA and miR-26b on the expression of CTGF and CCND1 in rPASMCs **A.** Determination and comparison of expression of rno-miR-26b between rPASMCs harvested from monocrotaline-treated and normal saline-treated rats (p<0.01); **B.** Determination and comparison of mRNA expression level of CTGF in rPASMCs harvested from the rats treated with normal saline, monocrotaline+NCshRNA, CTGF shRNA, CCND1 shRNA, and miR-26b (p<0.01); **C.** Determination and comparison of mRNA expression level of CCND1 in rPASMCs harvested from the rats treated with normal saline, monocrotaline+NCshRNA, CTGF shRNA, CCND1 shRNA, and miR-26b (p<0.01); **D.** Upper panel: Densitometry analysis of the western blotting results to show the relative protein levels of CCND1 in rPASMCs harvested from the rats treated with normal saline, monocrotaline+NCshRNA, CTGF shRNA, CCND1 shRNA, miR-26b; Lower panel: Results of western blot (p<0.01); **E.** Upper panel: Densitometry analysis of the western blotting results to show the relative protein levels of CTGF in rPASMCs harvested from the rats treated with normal saline, monocrotaline+NCshRNA, CTGF shRNA, CCND1 shRNA, miR-26b; Lower panel: Results of western blot (p<0.01). All experiments were repeated three times with three independent animals for each treatment group.

The microarray study was conducted and expression profiles were compared between rPASMCs isolated from monocrotaline-treated rats and the controls. No significant difference was noted between animal groups in this study regarding age and weight. Based on the evaluation of fold change (absolute fold change>1.6), 17 miRNAs were significantly differentially expressed in rPASMCs isolated from monocrotaline-treated rats compared with the controls with 11 up-regulated and 6 down-regulated (Table 1). The expression patterns were analyzed and compared between the two groups, and no pattern was identified specific to monocrotaline treatment. Furthermore, the most significantly down-regulated miRNAs, miR-26b, was selected for further functional analysis due to its well documented role in regulating human cell proliferation [30, 31], as well as the predicted function to suppress the expression of CTGF and CCND1(www.targetscan.org), upregulation of which have both been reported to contribute to the development of PAH in our *previous studies* [29, 32, 33]. The following confirmatory real-time PCR showed mRNA expression levels of rPASMCs isolated from monocrotaline-treated rats and the controls for selected miRNA (Figure 2A, *P<0.01), and a comparable change were identified compared with our precedent microarray experiment. The microRNA and their seed sequences were described in Figures 1B, 1C, and the 3′-UTR of CTGF and CCND1 were highly conserved, as presented in Figure 1A. We found that overexpression of miR-26b, but not the control mimics, substantially repressed the activity of luciferase fused with wild-type 3′-UTR of CTGF and CCND1, respectively, but had minimal effect on the luciferase activity fused with mutated 3′-UTR of CTGF or CCND1, as shown in Figures 1D, 1E (*P<0.01).

**Table 1:** Differentially expressed miRNAs identified in PASMCs harvested from monocrotaline-treated rats and the control

miRNA	Control mean	MCT-treated mean	Fold change
Upregulated miRNAs			
Rno-miR-218b	0.391322	1.943843	4.967371
Rno-miR-495	0.054737	0.227464	4.15556
Rno-miR-191a	2.102933	8.543625	4.062718
Rno-miR-21	0.695837	2.768848	3.97916
Rno-miR-16	0.173625	0.618484	3.56218
Rno-miR-382	1.238473	3.805432	3.07268
Rno-miR-425	0.264837	0.765413	2.890127
Rno-miR-375	2.432644	5.786544	2.378705
Rno-miR-190a	1.578766	3.456494	2.189365
Rno-miR-361-5p	0.674425	1.353569	2.006998
Rno-miR-762	2.355464	3.677948	1.561454
Downregulated miRNAs			
Rno-miR-26b	1.275893	0.256449	0.200995
Rno-miR-379	1.283746	0.468382	0.364856
Rno-miR-152	1.854748	0.648379	0.349578
Rno-miR-181a-2	2.578957	1.145384	0.444127
Rno-miR-30a	4.245663	2.047382	0.482229
Rno-miR-665	0.693625	0.385366	0.555582

### Treatment with monocrotaline significantly downregulated miR-26b expression in rats

Figure 2A showed that relative expression of miR-26b in the rPASMCs isolated from monocrotaline-treated rats was significantly down-regulated to about 20% compared with the control (*P<0.01). Consistent with our previous studies, and mRNA and protein expression levels of CTGF and CCND1 in PASMCs derived from monocrotaline-treated rats was significantly higher than the control, as evidenced by real-time PCR assay (Figures 2B, 2C, *P<0.01), western blot (Figures 2D, 2E, *P<0.01), and immunohistochemistry (Supplementary Figure S2A, S2B and Supplementary Figure S3A, S3B). In addition, monocrotaline treatment also significantly up-regulated the protein expression level of α-SM-actin in pulmonary vessels compared with the control (Supplementary Figure S4A, S4B, *P<0.01). Moreover, monocrotaline treatment directly led to approximately 500% increase in pulmonary vessel wall thickness compared with control (H&E staining, data not shown).

### Delivery of Nucleotides/EXGEN500 complex in vivo

Plasmids containing miR-26b, CTGF or CCND1 shRNA, or the control were mixed with EXGEN500/5% Glucose prior to intratracheal administration. Visual detection of GFP expression under fluorescence microscope in lung tissues resected from experimental animals was used to evaluate the distribution of intratracheally delivered miR or shRNAs, finding that the GFP was mainly detectable in pulmonary vessels, airways and mesenchyme (Supplementary Figure S5A-S5C). Additionally, rPASMCs were isolated from the pulmonary vessels and fractions of GFP positive cells were flow cytometrically estimated to be approximately 95% (Data not shown).

### shRNA-based or microRNA-based downregulation of CTGF and/or CCND1 expression in pulmonary vessels

Our previous studies have shown that downregulation of CTGF and CCND1 by intratracheally administered plasmid-based shRNA could significantly, but only partially, restore the monocrotaline-induced pulmonary artery remodeling by suppressing the target gene, respectively [28,29]. Considering the reports that the upregulation of CTGF and CCND1 substantially contributes to the development of pulmonary vascular remodeling, as well as the observation that miR-26b significantly suppresses CTGF and CCND1 expression, we next evaluated its effect on monocrotaline-induced pulmonary vascular remodeling, and determined the influence of miR-26b/EXGEN500 complex on the expression of CTGF and CCND1 in parallel with CTGF and CCND1 shRNAs in monocrotaline-treated rats. As shown in Figures 2B-2E, the treatment with miR-26b/EXGEN500 complex significantly attenuated both CTGF and CCND1 mRNA/protein expression in rat pulmonary vessels compared with the control, while the two shRNAs specifically downregulated its designed target with minimal effect on the other one. In addition, miR-26b almost completely restored the monocrotaline-induced up-regulation of CTGF and CCND1, while shRNA only partially lowered the expression of its designed target, as shown in Supplementary Figure S2 and S3. Meanwhile, the treatment with CTGF or CCND1 shRNA, and miR-26b significantly decreased the pulmonary vessel wall thickness (42%, 45%, 20% of monocrotaline-treated, respectively) in monocrotaline-treated rats (H&E staining, images not shown). Furthermore, we showed in this study that the miR-26b also significantly blocked monocrotaline-induced upregulation of α-SM-actin in rats, and the shRNAs could only partially restored it (Supplementary Figure S4). The results demonstrated that even though CTGF or CCND1 shRNA treatment successfully inhibited the pulmonary vascular remodeling induced by monocrotaline, but neither of them could completely restore the pulmonary artery remodeling induced by monocrotaline, while miR-26b could almost completely restore it.

### Flow cytometry analysis of the inhibitory effect of miR-26b on cell cycle progression in comparison with CTGF or CCND1 shRNAs in rPASMCs

**Figure 3:**
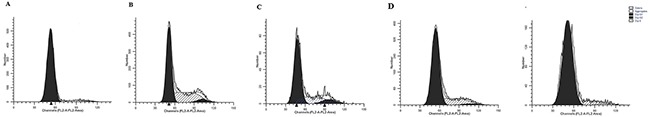
Effect of MCT, CTGF shRNA, CCND1 shRNA and miR-26b on the cell cycle status in rPASMCs **A.** Flow cytometric determination of cell cycle status in rPASMCs harvested from the rats treated with normal saline; **B.** Flow cytometric determination of cell cycle status in rPASMCs harvested from the rats treated with monocrotaline+NCshRNA; **C.** Flow cytometric determination of cell cycle status in rPASMCs harvested from the rats treated with monocrotaline and CTGF shRNA; **D.** Flow cytometric determination of cell cycle status in rPASMCs harvested from the rats treated with monocrotaline and CCND1 shRNA; **E.** Flow cytometric determination of cell cycle status in rPASMCs harvested from the rats treated with monocrotaline and rno-miR-26b. All experiments were repeated three times with three independent animals for each treatment group (N value=3).

Cell cycle analysis was performed to determine whether there was any cell cycle alteration in the rPASMCs in each group. The result showed that, compared with control group, the G1 phase percentage of rPASMCs treated with CTGF or CCND1 shRNA or miR-26b was increased from 56.82% to 65.55% (CTGF shRNA alone, P<0.01), 56.82% to 73.55% (CCND1 shRNA alone, P<0.01), and 56.82% to 87.00% (miR-26b alone, P<0.01), respectively, whereas the cells at the S phase decreased from 36.64% to 25.41% (CTGF shRNA alone, P<0.01), 36.64% to 23.70% (CCND1 shRNA alone, P<0.01), and 36.64% to 13.00% (miR-26b alone, P<0.01) (Figures 3A-3E). These results indicated that treatment with miR-26b substantially induced G1 cell cycle arrest in rPASMCs, and its inhibitory effect was stronger than that of in the groups treated with CTGF or CCND1 shRNA.

### In vitro analysis of the inhibitory effect of miR-26b on the expression of CTGF and CCND1 in comparison with CTGF or CCND1 specific siRNA in hPASMCs

**Figure 4:**
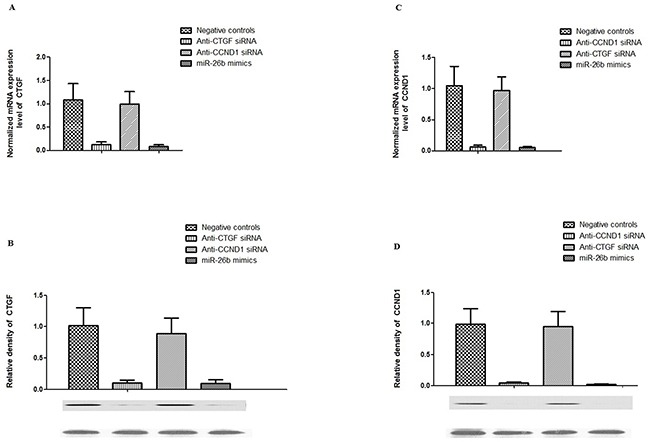
Effect of CTGF siRNA, CCND1 siRNA and miR-26b on the expression of CTGF and CCND1 in rPASMCs **A.** Effect of introduction of anti-CTGF siRNA, anti-CCND1 siRNA, has-miR-26b mimics and control on the mRNA expression level of CTGF in hPASMCs (p<0.01); **B.** lower panel: Effect of introduction of anti-CTGF siRNA, anti-CCND1 siRNA, has-miR-26b mimics and control on the protein expression level of CTGF in hPASMCs, as determined by western blot; upper panel: densitometric analysis of the western blot results underneath (p<0.01); **C.** Effect of introduction of anti-CTGF siRNA, anti-CCND1 siRNA, has-miR-26b mimics and control on the mRNA expression level of CCND1 in hPASMCs (p<0.01); **D.** lower panel: Effect of introduction of anti-CTGF siRNA, anti-CCND1 siRNA, has-miR-26b mimics and control on the protein expression level of CCND1 in hPASMCs, as determined by western blot; upper panel: densitometric analysis of the western blot results underneath (p<0.01). All experiments were repeated three times (N value=3).

To further investigate the therapeutic role of miR-26b in human tissues, we transfected hPASMCs with miR-26b mimics, CTGF or CCND1 specific siRNA, and examined the inhibitory effect of miR-26b on the expression levels of CTGF and CCND1 in hPASMCs, finding that the effect of specific siRNA was similar to that of miR-26b with respect to the inhibition of the expression of the designed target gene, whereas only miR-26b could suppressed the expression of both CTGF and CCND1 (Figure 4).

## DISCUSSION

Although miRNAs have been reported to be responsible for the regulation of numerous physiological and pathological processes, the role of miRNAs in the development of monocrotaline-induced pulmonary vascular remodeling and pulmonary hypertension has not been well characterized. Previously, Courboulin et al. [34] found that miR-204 is downregulated in PASMCs from patients with PAH and PASMCs from rats with monocrotaline-induced PAH. They showed that reconstitution of miR- 204 attenuated monocrotaline-induced PAH in rats. Caruso et al. [34] performed miRNA profiling and found that a number of miRNAs demonstrated significantly altered expressions in the lungs of rats that were exposed to hypoxia or were given monocrotaline. These data suggested that miRNAs participate in the pathogenesis of PAH in both patients and rodent models. In this study, the microarray study was conducted and expression profiles were compared between PASMCs isolated from monocrotaline-treated rats and the controls. Based on the evaluation of fold change (absolute fold change > 1.6), 17 miRNAs were significantly differentially expressed in rPASMCs collected from monocrotaline-treated rats compared with the controls with 11 upregulated and 6 downregulated (Table 1). By searching online microRNA databases, we identified both CTGF and CCND2 as potential targets of miR-26b, the most down-regulated one. Furthermore, we validated CTGF and CCND1 as effective targets of miR-26b by using a luciferase reporter system.

CTGF, a 38 kDa cysteine-rich protein, is a member of the CCN family. It was firstly identified in the conditioned medium of human umbilical vein endothelia cells [35], and has been found to be involved in the regulation of cell adhesion, migration, proliferation, and extracellular matrix (ECM) synthesis in a variety of cell types, including vascular endothelial cells, fibroblasts, osteoblastic cells, chondrocytic cells, and smooth muscle cells [6-10]. Previous studies have documented that CTGF plays an important role in airway remodeling [36, 37]. Cyclin D1 is a protein that is encoded by CCND1 gene, and it belongs to the highly conserved cyclin family. It has been generally agreed that cyclins, including D type cyclins, may function together with their CDK partners to play a major role in the fine tune of cell cycle control [38]. In this study, we found that overexpression of miR-26b, but not the control, substantially repressed the activity of luciferase fused with 3′-UTR of CTGF and CCND1 respectively, but had minimal effect on the luciferase activity fused with mutated 3′-UTRs, as shown in Figure 1. In addition, we found that relative expression of miR-26b in the rPASMCs isolated from monocrotaline-treated rats was significantly downregulated compared with the control. Consistent with our previous studies, and mRNA and protein expression levels of CTGF and CCND1 in rPASMCs derived from monocrotaline-treated rats was significantly higher than the control (Figure 2).

MiR-26b has been shown to be able to suppress the proliferation of human multiple myeloma cells via targeting a number of candidate genes including CTGF [39]; Meanwhile, Yang et al demonstrated in their study about human breast cancer that miR-26b inhibited breast cancer progression through modulating Fra-1 proto-oncogene [27]. In our previous study, we showed plasmid-based CTGF specific shRNA decelerated the proliferation of rPASMCs and attenuate monocrotaline-induced pulmonary artery remodeling via suppressing the expression of CTGF [29]. In another study, we demonstrated that knockdown of CCND1 with plasmid-based shRNA could significantly suppress the rPASMCs proliferation induced by smoke extract in vitro and ameliorated the pulmonary vascular remodeling in rats exposed to cigarette smoke in vivo [28]. In the following study, we confirmed the aforementioned results, and proved the that CCND1 mediated the effect of CTGF by promoting G1/S transition in the cell cycle, explaining partially how CTGF enhanced rPASMCs proliferation and contributed to the development of pulmonary vascular remodeling in rats [40]. Considering the previous reports that miR-26b inhibited human cell proliferation, and the observation that miR-26b substantially suppressed the expression of CTGF and CCND1, we subsequently evaluated the efficacy of miR-26b in the treatment of monocrotaline-induced pulmonary artery remodeling in parallel with CTGF or CCND1 shRNA, and found miR-26b, CTGF shRNA, or CCND1 shRNA significantly decreased the pulmonary vessel wall thickness (42%, 45%, 20%, of monocrotaline treated, respectively) in monocrotaline-treated rats. The results demonstrated that even though CTGF or CCND1 shRNA treatment successfully attenuated the pulmonary vascular remodeling induced by monocrotaline, nevertheless neither of them could completely restore the pulmonary artery remodeling, which can be done by introduction of miR-26b alone. We reasoned that miR-26b may exert protective effect via inhibiting both CTGF and CCND1, or possibly involved some other PAH-related genes which are not necessarily a complete match, and therefore not predictable by the online target predicting tool. STAT3 has also been reported to be regulator of CTGF [41]. We examined the expression of STAT3 in each treatment group, and found no difference among those groups (data not shown).

DNA or RNA/EXGEN500 is a nucleic acids delivery method with high transfection efficiency and low toxicity, and optimized PEG shielding has been showed to be able to promote intratracheal gene introduction via limiting inflammatory responses in lungs [42]. As a newly developed therapeutic molecule, microRNA has been reported to be intratracheally given to alter the expression of multiple target genes in lungs and successfully treat pulmonary disease [43]. In our previous work, we have steadily showed that plasmid-based shRNA/EXGEN500 could be intratracheally delivered to lung with highest expression detected in pulmonary artery walls and successfully treat the modeled lung disease by knocking-down the target gene in experimental animals and the efficacy was satisfactory. In this study, a similar delivery efficiency and a better efficacy of miR-26b/EXGEN500 were exhibited in the treatment of same disease, and this may shed a light on the development of therapeutic tool in treat PAH in human.

Taken together, we showed that PASMCs from monocrotaline-induced pulmonary artery remodeling are different from the normal controls in their microRNA repertoire, and miR-26b, a microRNA significantly downregulated in pulmonary artery remodeling, contributes to the development of PAH via releasing the inhibition of its two target genes, CTGF and CCND1, both of which have been repeatedly reported to be involved in the pathogenesis of the disease. The discovery of the downregulation of miR-26b in pulmonary artery remodeling, as well as its regulation of its target genes, CTGF and CCND1, may have important implications in our understanding of the molecular mechanisms underlying PAH, and may also lead to the development of new therapeutic interventions in this devastating and life-threatening disease.

## MATERIALS AND METHODS

### Total RNA isolation

The rPASMCs harvested from monocrotaline-treat rats or the control were pooled together, and total RNA was extracted using Trizol reagent (Invitrogen, Carlsbad, CA, USA) according to the manufacturer's instructions with little modification. The purity of the isolated RNA was determined using a spectrophotometer (Jenway Ltd, Essex, UK). RNA integrity was confirmed by electrophoresis in an agarose gel.

### miRNA microarray analysis

5ug of total RNA from rPASMCs (monocrotaline-treated or the control) was used in the microarray expression profiling assay. The miRNAs were fluorescently labeled using MiRCURYTM Array Labeling kit (Exiqon, Vedbaek, Denmark) before hybridized to the miRNA microarray chip (Exiqon, Vedbaek, Denmark). A GenePix 4000B laser scanner was used to collect hybridization data, and the images were digitized and analyzed by GenePix 4.0 software (Axon Instruments, Foster City, CA, USA).

### Luciferase reporter assay

The rat CTGF and CCND1 wild-type 3′-UTRs were PCR amplified and cloned into a modified version of pcDNA3.1(+) that contained a firefly luciferase reporter gene, at a position downstream of the luciferase reporter. The vectors were named wild-type 3′UTRs of CTGF or CCND1, respectively. Site-directed mutagenesis of the miRNAs binding sites in the 3′UTRs was performed using Site-Directed Mutagenesis Kit (SBS Genetech, Beijing, China) and named as mutant 3′UTRs, as shown in Figure 1. rPASMCs grown in a 48-well plate were co-transfected with 400 ng of either individual miR-26b, 40 ng of the firefly luciferase reporter plasmid including the 3′-UTR of the target gene, and 4 ng of pRL-TK, a plasmid expressing rellina luciferase (Promega, Madison, WI, USA). After 24 h, the cells were collected, and the luciferase reporter assay was performed in TD-20/20 luminometer (Turner Biosystems, Sunnyvale, CA). The primers for cloning and site-directed mutagenesis were described in Supplementary Figure S1.

### Plasmid vectors construction

The plasmid-based shRNA vector was constructed as previously described [32]. Briefly, the oligonucleotides for CTGF or CCND1 shRNAs, miR-26b or the negative control were synthesized chemically or PCR-amplified, and linked into the pGPU6/GFP vector which contains a mouse U6 RNA polymerase III promoter. The oligonucleotide sequences for CTGF or CCND1 shRNAs, or the primer set for miR-26b were described in Supplementary Figure S1. Direct Sanger sequencing was used to confirm the sequences of inserts prior to the following functional analysis.

### Animal treatment and gene delivery

All animal experiments were performed according to the protocols approved by the Institutional Animal Care and Use Committee of Anhui medical university. Fifty male healthy Sprague–Dawley (SD) rats of SPF degree, weighing 200–250 g, 10 weeks old, were provided by Experimental Animal Center of Anhui medical university, and divided equally into five groups (Group I: Treated with normal saline (as animal model establishment control); Group II: Treated with monocrotaline (Sigma–Aldrich, St. Louis, MO, USA) and negative control shRNA (as therapeutic control); Group III: Treated with monocrotaline and CTGF shRNA; Group IV: Treated with monocrotaline and CCND1 shRNA; Group V: Treated with monocrotaline and miR-26b (No difference was identified regarding the observed parameters such as cell proliferation, vascular thickness, the expression of CTGF, CCND1,α-SM actin, or cell cycle progression between the rats treated with or without normal saline, and between the rats treated with monocrotaline and monocrotaline+ NCshRNA (Negative control shRNA), as presented in our previous studies, so we narrowed down the original seven groups to five experimental groups in this study). A single injection of 60 mg/kg monocrotaline was subcutaneously administered to establish the model of pulmonary vascular remodeling in rats, as described previously [33, 44]. Rats were anaesthetized with IP sodium pentobarbital (40mg/kg), and the animals were sacrificed by exsanguination.

For gene delivery in vivo, plasmid-based CTGF or CCND1 shRNA, miR-26b, or its negative control were mixed with EXGEN500 and 5% glucose (Fermentas Biotechnology Co. Ltd, Burlington, Ontario, Canada). Rats were intratracheally treated with Nucleotides/EXGEN500 complex (at ratio of 100 μg/330 μl) every week.

### Tissue preparation and morphometric analysis of pulmonary vessels

After three weeks, all rats were sacrificed by exsanguination and the lungs were harvested. Right lungs were fixed in 4% paraformaldehyde overnight. Fixed tissues were embedded in paraffin and cut into 5μm thick sections. Explants of intra-pulmonary arteries were isolated from left lungs and stored at −80°C for future use.

Sections were deparaffinized and stained with hematoxylin and eosin (H&E). Then the H&E-stained tissue sections were observed under a light microscope and assessment of vascular morphology was carried out. The measurement was limited to small arteries (≤50μm in external diameter) adjacent to the alveolar ducts. In each section, at least ten vessels were measured. The pulmonary vessel wall thickness was expressed as the percentage of the external diameter [(2× measured wall thickness/external diameter) ×100].

### Immunochemistry staining

Histologic sections were deparaffinized with xylene and ethanol. Antigen retrieval was performed using 10 mM citrate buffer, pH 6.0. The sections were blocked with 10% normal goat serum for 30 min, followed by an overnight incubation at 4°C with rabbit polyclonal antibody against CTGF (1:200) (Santa Cruz, CA, USA), or CCND1 (1:200) (Santa Cruz, CA, USA). A negative control, replacing the primary antibody with goat IgG at the same concentration, was included. Biotinylated goat anti-rabbit (diluted 1:200) (Santa Cruz, CA, USA) was followed by an incubation with streptavidin-peroxidase conjugate.

### Assessment of pulmonary vascular remodeling

Immunohistochemical staining with anti-α-smooth muscle actin antibody (Santa Cruz, CA, USA) was performed for additional evaluation of pulmonary vascular remodeling. All small intrapulmonary arteries (≤50 μm in external diameter) adjacent to the alveolar ducts were analyzed. Each vessel was categorized as nonmuscularized (actin staining <25% of the circumference), partially muscularized (actin staining 25–75% of the circumference) and fully muscularized (actin staining >75% of the circumference). The fully muscularized vessels were calculated and expressed as the percentage of total small intrapulmonary arteries. All morphometric measurements were performed by 2 independent researchers operating in a blinded manner. Interobserver difference in the measurements was <5%.

### Western blot analysis

Total protein was extracted, and total protein concentration was determined by the Bradford method using Bradford reagent (Bio-Rad, Hercules, CA, USA), and then separated by 10% sodium dodecyl sulfate–polyacrylamide gel electrophoresis (SDS–PAGE). The proteins were subsequently transferred to polyvinylidene difluoride membranes and blocked for 2 hours in PBS with 0.1% Tween containing 5% nonfat dried milk. The membranes were then incubated with primary antibody, CTGF (1:500), or CCND1 (1:500) overnight and then washed three times with the buffer. The membranes were incubated with horseradish peroxidase-conjugated goat anti-rabbit IgG secondary antibody (1:10,000) for 1 hour, and then washed three times. Immunoreactivity was detected using an enhanced chemiluminescence Western blotting detection kit (Pierce, Rockford, IL, USA) according to the manufacturer's instructions. Immunoblots were scanned using a GS-800 densitometer and protein bands were quantified with Quantity One software (Bio-Rad Laboratories, Hercules, CA, USA). GAPDH was used to normalize the results prior to further densitometrical analysis.

### Tissue preparation and morphometric analysis of pulmonary vessels

Each tissue specimen was fixed in 4% paraformaldehyde. After dehydration, it was embedded in paraffin and selected for 5mm-thick serial sectioning. Small intrapulmonary artery branches were isolated under optical microscope, and then the endothelial surface and the surrounding adventitia were carefully dissected, the medial layer was immediately stored at −80°C.

Sections were deparaffinized, H&E stained, immunostained and observed under a light microscope. For each patient, five arteries with external diameter between 50-500mm and structurally integral laminas were randomly selected and measured. The pulmonary vessel wall thickness was expressed as the percentage of the external diameter [(2×measured wall thickness/external diameter) ×100].

### Cell culture

Segments of pulmonary artery (50-500mm external diameter) were incubated in Hanks' solution containing collagenase (1.5 mg/mL) for 20 minutes. After incubation, a thin layer of adventitia was carefully stripped off with a fineforcep, and the endothelium was removed by gentle scratching of the intimal surface with a surgical blade. The remaining smooth muscle was then digested with collagenase (2.0 mg/mL) and elastase (0.5 mg/mL) for 35–45 minutes at 37°C. Cells were cultured in Dulbecco's modified Eagle's Medium (DMEM) (Life Technologies Inc., USA) containing 10% fetal bovine serum, penicillin (100U/mL), and streptomycin (100 mg/mL) and cultured in a humidified incubator at 37°C. The cells were passaged by trypsinization with 0.05% trypsin–EDTA and used for experiments at passages 3-8.

### Cell cycle analysis

To estimate the proportions of cells in different phases of the cell cycle, cellular DNA contents were measured by flow cytometry. After treatments, cells were harvested, washed twice with cold PBS, and then fixed overnight at −20°C in 70% ethanol. Immediately before flow cytometry, the cells were resuspended in PBS containing PI (50 m g/ml) and DNase-free RNase (10 m g/ml). Flow cytometry was performed using a FACScalibur (Becton Dickinson, San Diego, CA, USA) system with CELLquest software. The percentages of cells in different phases of the cell cycle were determined using the ModFit software.

### RNA extraction and quantitative real-time PCR

Total RNA was extracted by using Trizol according to the manufacturer's protocol. Quantitative real-time PCR was performed using the SYBR Green Realtime PCR Master Mix (QPK-201, Toyobo, Japan) with the Light Cycler Instrument (Roche Diagnostics Corp., Basel, Suisse, Swiss). Each sample was examined in triplicate and the amounts of the PCR products produced were normalized to that of GAPDH which served as internal control. The primers used in the study were described in Supplementary Figure 1. Relative transcript levels of each gene were normalized using GAPDH as the housekeeping gene.

### siRNA transfection

The siRNA sequences of human CTGF or CCND1 were described in Supplementary Figure 1. For transfection, cells were seeded into plates, incubated overnight, and then transfected using Lipofectamine 2000 transfection reagent according to the manufacturer's instructions at 40–60% cell confluence.

### Statistical analysis

The experiments were repeated at least three times, and all results were expressed as mean (M) ± standard deviation (SD). Statistical analysis was carried out using one-way ANOVA (for multiple-group comparison) followed by the least significant difference (LSD) test or student t-test (for two-group comparison) with the computer software SPSS 12.0 (SPSS, Chicago, IL, USA). For all tests, data were considered statistically significant when P < 0.05.

Raw data of the miRNA microarray were normalized by GenePix 4.0 software (Axon Instruments), and median centered using the Bioconductor package (www.bioconductor.org). SAM software (http://www.stat.stanford.edu/tibs.SAM) was used to determine the differentially expressed miRNAs in the PASMCs collected from monocrotaline-treated rats compared with the control, among which we listed only those genes with significant differential expression of 1.6-fold higher or lower.

## SUPPLEMENTARY MATERIALS FIGURES



## Abbreviations

PAH: Pulmonary artery hypertension
PASMCs: pulmonary artery smooth muscle cells
CTGF: connective tissue growth factor
CCND1: cyclin D1
ECM: extracellular matrix
rPASMCs: rat PASMCs
hPASMCs: human PASMCs
UTR: untranslated region
miRNA: MicroRNA
SDS–PAGE: sodium dodecyl sulfate–polyacrylamide gel electrophoresis
H&E: hematoxylin and eosin
DMEM: Dulbecco's modified Eagle's Medium
NCshRNA: Negative control shRNA
