# Supplementary material for: MicroRNA-26b attenuates monocrotaline-induced pulmonary vascular remodeling via targeting connective tissue growth factor (CTGF) and cyclin D1 (CCND1)
Source: Oncotarget. 2016 Jun 17;7(45):72746–57. doi: 10.18632/oncotarget.10125 (PMC5341941; doi:10.18632/oncotarget.10125)
Supplement: Supplementary file 1 [file oncotarget-07-72746-s001.pdf]

MicroRNA-26b attenuates monocrotaline-induced pulmonary vascular remodeling via targeting connective tissue growth factor (CTGF) and cyclin D1 (CCND1)

SUPPLEMENTARY FIGURES

Name	Sequences
**Real-time**<br>**PCR Primers**<br>Has-miR-26b	5'-CGCCCTGTTCTCCATTACTT-3'
	5'-CCAGTGCAGGG TCCGAGGT-3'
Rno-miR-26b	5'-GGGGTTCAAGTAATTCAGG-3'
	5'-CAGTGCGTGTCGTGGA-3'
CTGF (human)	5'-ACTATGATTAGAGCCAACTG-3'
	5'-TGTTCTCTTCCAGGTCAG-3'
CCND1 (human)	5'-CGTGGGCTCTAAGATGAAGG-3'
	5'-TGCGGATGATCTGTTTGTTC-3'
GADPH (human)	5'-GAAGGTGAAGGTCGGAGTC-3'
	5'-GAAGATGGTGATGGGATTTC-3'
CTGF (rat)	5'-CGTTTGTGCCTATTGTTCTTGTT-3
	5'-TGATCCATTGCTTTACCGTCTAC-3
CCND1 (rat)	(5'-TGTTCGTGGCCTCTAAGATGAAG-3'
	5'-GGAAGTGTTCGATGAAATCGTG-3'
GADPH (rat)	5'-GACTACCTCATGAAGATCCTG-3
	5'-CATAGAGGTCTTTACGGATGT-3'
**Subcloning**<br>**Primers**<br>CTGF 3'UTR	5'-CGGGTACCTAAAGCCAGGGAGTAAGGGACACGAACTCA-3'<br>5'-<br>ATAAGAATGCGGCCGCCATATAAAAAATATATATACTTTATTTTC<br>AAC-3'
CCND1 3'UTR	5'-<br>CGGGTACCTGAGGGCCACCGGGCAGGCGGGAGTCACCAA<br>G-3'
**Mutagenesis**<br>**Primers**<br>CTGF 3'UTR mutant	5'-<br>ATAAGAATGCGGCCGCTGGGATTTTACCAATTTTATTTCTAGA-<br>3'
CCND1 3'UTR<br>mutant	5'-<br>ACGAACTCATTTAGACTATATGAACTTCTGAGTTACATCTCA<br>TTTT-3'<br>5'-<br>AAAATGAGATGTAACTCAGAAGTTCATATAGTCTAAATGAGT<br>TCGT-3'
**shRNA**<br>CTGF (rat)	5'-TTGGAAGTAGGGACCGTGATGCTTAATGAACTT<br>CAAGGGGATTCAGGACGAC-3'<br>5'-GTCGTCCTGAATCCCCTTGAAGTTCATTAAGCATCA<br>CGGTCCCTACTTCCAA-3'
CCND1 (rat)	

Supplementary Figure S1: Sequences information on shRNA, microRNA, and primer sets for subcloning, mutagenesis, and real-time PCR. ***(Continued)***

	5’-CACCGCAATACCTTCTGCAGGCTGGATTCAAGAG
	ATCCAGCCTGCAGAAGGTATTGTTTTTTG-3’
**siRNA**	5’-GATCCAAAAAACAATACCTTCTGCAGGCTGGATCTCT
CTGF (human)	TGAATCCAGCCTGCAGAAGGTATTGC-3’
	5’- CACCGCCGAGAAGTTG TGCATCTATTCA
CCND1 (human)	AGAGATAGATGCACAACTTCTCGGTTTTTTG-3’
	5’-GATCCAAAAAACCGAGAAGTTGTGCATCTATC
	TCTTGAATAGATGCACAACTTCTCGGC-3’
**microRNA**	
**in plasmid**	
Rno-miR-26b	5’-CATCGATAGCATCTTATACGA-3’
	5’- TCGTATAAGATGCTATCGATG-3’
	5’-TCTGAGGGCGCCAGGCAGGCGG-3’
**microRNA**	5’-CCGCCTGCCTGGCGCCCTCAGA-3’
**mimics**	
Has-miR-26b	
	Forward: ’-CGGAAGACGGACTACCTGGTACTCCAGAAAT-’
	Reverse: ’-ATGGATCCAGTTGGTCCCACCTCACCTAG-’
	5’- UUCAAGUAAUUCAGGAUAGGU-3’

Supplementary Figure S1: ***(Continued)*** **Sequences information on shRNA, microRNA, and primer sets for subcloning, mutagenesis, and real-time PCR.**

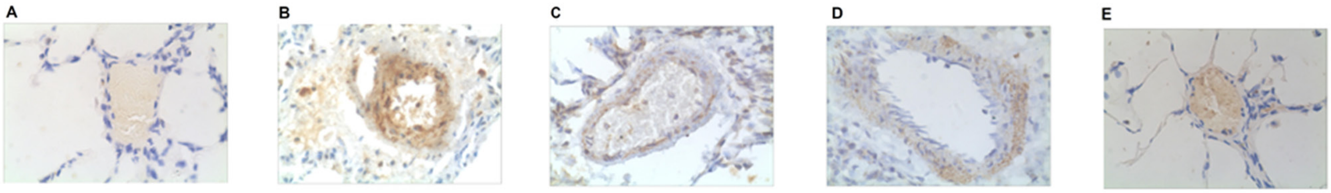

Supplementary Figure S2: Effect of MCT, CTGF shRNA, CCND1 shRNA and miR-26b on the expression of CTGF in rPASMCs. Monocrotaline treatment substantially up-regulated the expression of CTGF **B.** compared with the control that’s treated with normal saline **A.** The treatment with CTGF shRNA **C.** and rno-miR-26b **E.** but not CCND1 shRNA **D.** significantly lowered the expression of CTGF induced by monocrotaline; Lung histologic sections were stained with anti-CTGF antibody. All experiments were repeated three times (N value=3).

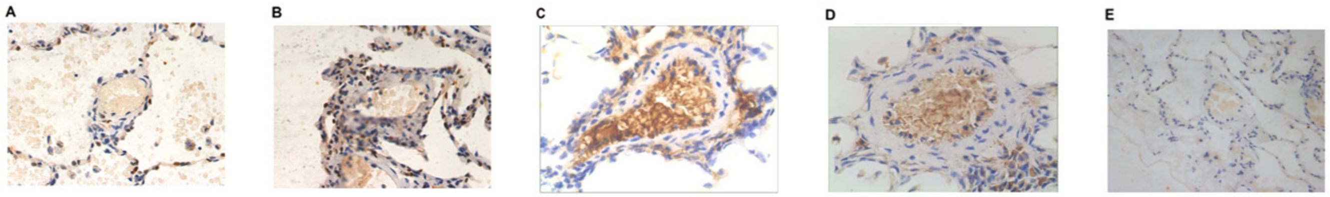

Supplementary Figure S3: Effect of MCT, CTGF shRNA, CCND1 shRNA and miR-26b on the expression of CCND1 in rPASMCs. Monocrotaline treatment substantially up-regulated the expression of CCND1 **B.** compared with the control that's treated with normal saline **A.** The treatment with CCND1 shRNA **D.** and rno-miR-26b, but not CTGF shRNA **C.** significantly lowered the expression of CCND1 induced by monocrotaline; Lung histologic sections were stained with anti-CCND1 antibody. All experiments were repeated three times (N value=3).

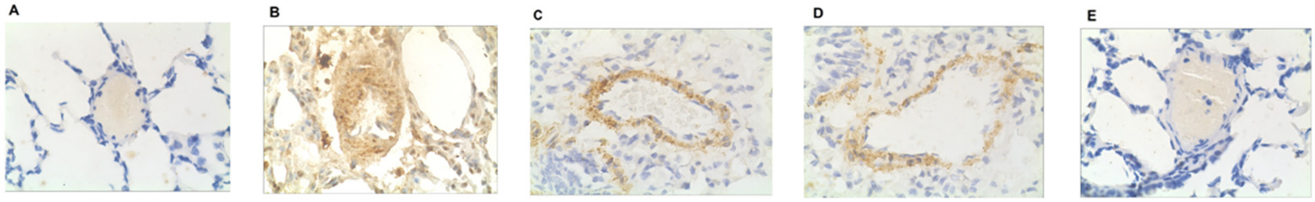

Supplementary Figure S4: Effect of MCT, CTGF shRNA, CCND1 shRNA and miR-26b on the expression of β-SM-actin in rPASMCs. Monocrotaline treatment substantially upregulated the expression of β-SM-actin **B.** compared with the control that's treated with normal saline **A.** The treatment with CCND1 shRNA **D.** and CTGF shRNA **C.** significantly, but only partially, lowered the expression of β-SM-actin induced by monocrotaline, while rno-miR-26b could almost completely restore it **E.** Lung histologic sections were stained with anti-β-SM-actin antibody. All experiments were repeated three times (N value=3).

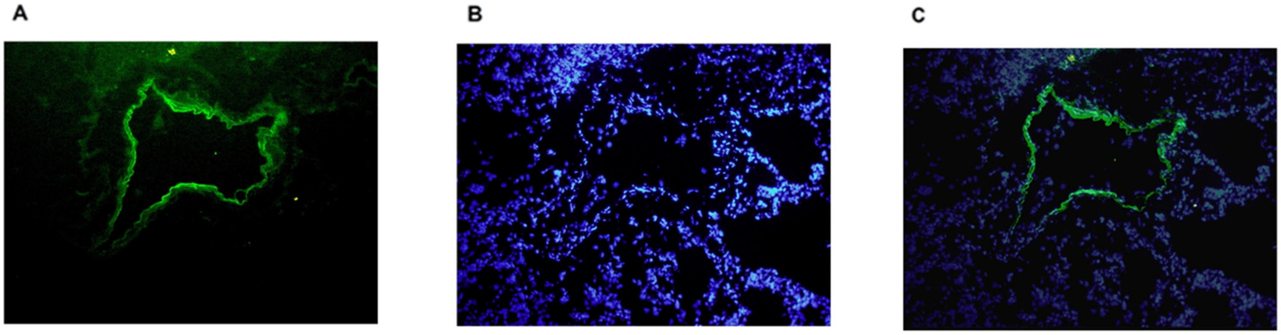

Supplementary Figure S5: Evaluation of delivery efficiency. A. Fluorescence microscopic detection of GFP expression in pulmonary smooth muscle isolated from pulmonary arteries of experimental animals was used to evaluate the distribution of intratracheally delivered shRNA, showing that the GFP was mainly expressed in pulmonary vessels, airways and mesenchyme; **B.** DAPI staining of the same section of lung tissue; **C.** Overlapped show of both A and B. All experiments were repeated three times (N value=3).
